# Functional interactions between nitrite reductase and nitric oxide reductase from *Paracoccus denitrificans*

**DOI:** 10.1038/s41598-019-53553-z

**Published:** 2019-11-21

**Authors:** Ingrid Albertsson, Johannes Sjöholm, Josy ter Beek, Nicholas J. Watmough, Jerker Widengren, Pia Ädelroth

**Affiliations:** 1 Department of Biochemistry and Biophysics, Stockholm University, Svante Arrhenius väg 16C, SE-106 91 Stockholm, Sweden; 2 Experimental Biomolecular Physics, Department of Applied Physics, Royal Institute of Technology (KTH), SE-106 91 Stockholm, Sweden; 3 Present Address: Department of Medical Biochemistry and Biophysics, Umeå University, Umeå, SE-90187 Sweden; 4 School of Biological Sciences, University of East Anglia, Norwich Research Park, Norwich, NR4 7TJ UK

**Keywords:** Oxidoreductases, Kinetics

## Abstract

Denitrification is a microbial pathway that constitutes an important part of the nitrogen cycle on earth. Denitrifying organisms use nitrate as a terminal electron acceptor and reduce it stepwise to nitrogen gas, a process that produces the toxic nitric oxide (NO) molecule as an intermediate. In this work, we have investigated the possible functional interaction between the enzyme that produces NO; the *cd*_1_ nitrite reductase (*cd*_1_NiR) and the enzyme that reduces NO; the *c*-type nitric oxide reductase (*c*NOR), from the model soil bacterium *P. denitrificans*. Such an interaction was observed previously between purified components from *P. aeruginosa* and could help channeling the NO (directly from the site of formation to the side of reduction), in order to protect the cell from this toxic intermediate. We find that electron donation to *c*NOR is inhibited in the presence of *cd*_1_NiR, presumably because *cd*_1_NiR binds *c*NOR at the same location as the electron donor. We further find that the presence of *c*NOR influences the dimerization of *cd*_1_NiR. Overall, although we find no evidence for a high-affinity, constant interaction between the two enzymes, our data supports transient interactions between *cd*_1_NiR and *c*NOR that influence enzymatic properties of *c*NOR and oligomerization properties of *cd*_1_NiR. We speculate that this could be of particular importance *in vivo* during metabolic switches between aerobic and denitrifying conditions.

## Introduction

Denitrification is an anaerobic process in which nitrate (NO_3_^−^) is reduced stepwise to nitrogen gas (N_2_) via the intermediates nitrite, nitric oxide and nitrous oxide. There is widespread interest in denitrification because it limits the amount of nitrogen available to crops by decreasing the amount of nitrate and nitrite in the soil and because incomplete denitrification yields nitrous oxide which is a potent green-house gas. In *Paracoccus (P.) denitrifi*cans, a model organism for both aerobic respiration and denitrification, the enzymes that catalyze these reactions are: nitrate reductase (NAR), reducing nitrate to nitrite, nitrite reductase (NiR) which reduces nitrite to nitric oxide, nitric oxide reductase (NOR), reducing nitric oxide to nitrous oxide and finally nitrous oxide reductase (N_2_OR), which reduces nitrous oxide to nitrogen gas (for a review on denitrification enzymes, see^1^).

The stepwise reduction of nitrate requires the product of one enzyme to be the substrate for the next enzyme in the pathway and as a consequence the expression of all four enzymes should be coordinated and regulated in such way that the concentrations of nitrite and nitric oxide are kept at concentrations that are not toxic to the cell^2^. Lethal nitric oxide concentrations have been shown to vary between organisms, with some bacteria such as *Agrobacterium tumefaciens* accumulating µM NO concentrations during rapid switches between oxic and anoxic conditions, but in *P. denitrificans* nitric oxide is kept at (or below) ~30 nM^3^.

The enzyme catalyzing the reduction of nitrite to nitric oxide (NO_2_^−^ + e^−^ + 2 H^+^ →NO + H_2_O) in *P. denitrificans* is cytochrome *cd*_1_ nitrite reductase (*cd*_1_NiR), a soluble protein located in the periplasm (for a recent review on *cd*_1_NiR and nitrite, see^4^). The almost identical (97% sequence identity) and well characterized *cd*_1_NiR from *Paracoccus pantotrophus* is purified^5^ and crystallized as a dimer^6^. Each *cd*_1_NiR monomer consists of one small heme *c* domain and one large *d*_1_ domain, where NO_2_^−^ reduction takes place. The heme *c* domain receives electrons from one of two soluble donors; either cytochrome *c*^550^ or the copper protein pseudoazurin^7^. The heme *d*_1_ in the catalytic domain has an unusual ability (as compared to other hemes) to rapidly release NO, thereby lowering the degree to which the *cd*_1_NiR enzyme activity is inhibited by its product NO^8^.

The well-characterized *cd*_1_NiRs from *P. pantotrophus* and *Pseudomonas (Ps) aeruginosa* (see e.g.^9^ and^4^) have many properties in common including a similar overall fold especially in the larger, catalytic *d*_1_ domain. They also use similar electron donors; a soluble *c* cytochrome or a blue copper protein. However, there are also striking differences, such as the ‘domain swapping’ that occurs only in the *Ps. aeruginosa cd*_1_NiR dimer, where the N-terminal arm (in the cyt. *c* domain) of one monomer crosses over to interact with the *d*_1_ domain of the second monomer.

Nitric oxide, produced from *cd*_1_NiR, is further reduced to nitrous oxide (2NO + 2e^−^ + 2 H^+^ →N_2_O + H_2_O), by nitric oxide reductase (NOR). NORs are members of the heme-copper oxidase (HCuO) superfamily. This superfamily (comprising the cytochrome *c* oxidase in mitochondria) is large and diverse and some of its members are capable of NO-reduction^10–12^, and all that have been investigated also show that the physiological O_2_-reduction reaction is inhibited by NO (reviewed in^13,14^, see also^15^), an effect which is linked to the use of NO as a signaling molecule in mammals^16^.

The NOR from *P. denitrificans* is a cytochrome *c*-dependent NOR (*c*NOR) that, as purified, is composed of two subunits; NorB and NorC. The NorB is an integral membrane protein and harbors a low-spin heme *b* and the active site, composed of a high-spin heme *b*_3_ and a non-heme iron, Fe_B_. NorC is membrane-anchored and contains a periplasmic heme *c*, which receives electrons from soluble donors such as cytochrome *c* or pseudoazurin (the same as for *cd*_1_NiR). The enzyme uses protons and electrons from the same side of the membrane (periplasmic, see^17,18^) and is thus non-electrogenic^19,20^, which differs from the O_2_-reducing HCuOs. The crystal structure of the *c*NOR from *P. aeruginosa* supports this as putative proton transfer pathways are only found leading from the periplasm into the active site^21^.

**Figure 1:**
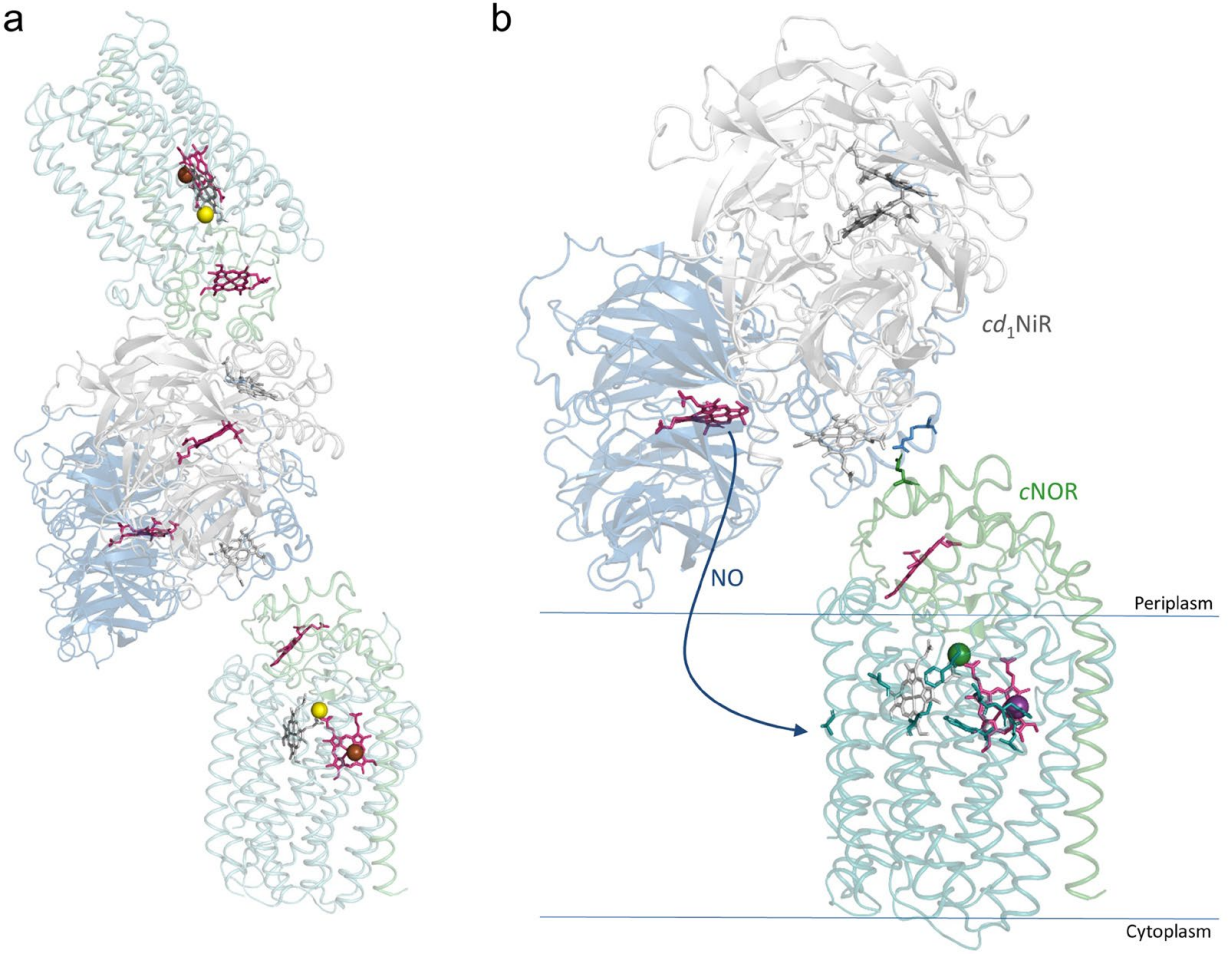
Structure of the co-complex of the *P. aeruginosa c*NOR and the *cd*_1_NiR dimer (PDB ID: 5GUW^24^). (**a**) The full 2:2 structure with the two *c*NOR molecules in light green (NorC) and teal (NorB) and the *cd*_1_NiR dimer in blue/gray. (**b**) Enlargement of the co-complex interaction area for *cd*_1_NiR and a single *c*NOR, with the interaction between Arg-71 (*cd*_1_NiR, blue stick) and Glu-119 (*c*NOR, green stick) shown. Also shown are schematic outlines of the cytoplasmic membrane in which *c*NOR sits and the path for NO from the release from the *d*_1_ heme of *cd*_1_NiR (pink) into the membrane from which it would travel through the suggested gas channel (indicated by green sticks) to the active site heme *b*_3_ (pink) in *c*NOR^25^. Also highlighted is the initial electron-accepting heme *c* in NorC (pink), other heme groups in grey.

Respiratory chain complexes in mitochondria commonly form higher-order complexes, so-called supercomplexes. Such supercomplexes have also been found in bacteria (see e.g.^22,23^), but the functional advantage of them is not always fully understood. Recently, the crystal structure of a complex between separately purified *cd*_1_NiR and *c*NOR from *Ps. aeruginosa* was presented^24^. The complex has a 2:2 stoichiometry (dimer of *cd*_1_NiR with two monomers of *c*NOR), and the interaction was suggested to be present also under native conditions, but then in a 2:1 stoichiometry since the membrane-location of *c*NOR is not compatible with the 2:2 complex observed. Such a *cd*_1_NiR-*c*NOR complex could confer advantages *in vivo* as the toxic NO molecule would, instead of being released into the periplasmic solution, rather be ‘channeled’ into the membrane in which it is more soluble. From the membrane, NO could directly enter the gas channel suggested for *c*NOR^21,25^, see Fig. 1.

The aim of this work was to determine whether the *P. denitrificans cd*_1_NiR and *c*NOR form a molecular complex *in vivo* and/or *in vitro* and to study potential functional interactions *in vitro*. To this end we investigated the localization of *cd*_1_NiR in *P. denitrificans*, and we also used the *c*NOR catalyzed reaction as an *in vitro* ‘handle’ to report on a possible complex with *cd*_1_NiR. We also used fluorescence spectroscopy to investigate *cd*_1_NiR dimerization and the interactions of *cd*_1_NiR with artificial and native membranes as well as with *c*NOR. Our data implies interference from *cd*_1_NiR binding on electron donation to *c*NOR, consistent with an overlapping interaction surface. This effect of *cd*_1_NiR on *c*NOR activity shows a titration profile consistent with an interaction primarily with a single *cd*_1_NiR monomer. Our fluorescence data is consistent with this dimerization occurring in the relevant concentration range (20–40 nM *cd*_1_NiR). However, we could not observe any clear long-lived high-affinity binding between *cd*_1_NiR and *c*NOR going beyond the rather high affinity *cd*_1_NiR showed to artificial membranes, nor could we observe a large fraction of the *cd*_1_NiR associated with the membrane-bound *c*NOR in *P. denitrificans* membranes. Potential *in vivo* consequences of our results are discussed.

## Results

### The influence of *cd*_1_NiR on catalytic activity of *c*NOR

**Figure 2:**
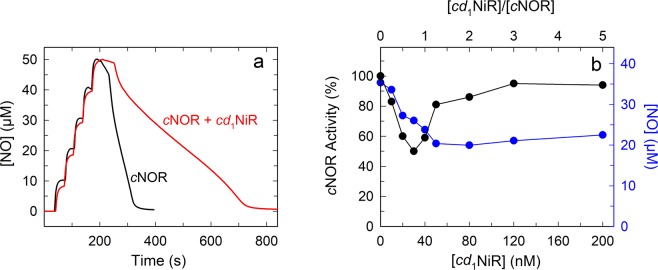
The inhibitory effect of *cd*_1_NiR on *c*NOR catalysis. (**a**) NO reduction profile of *P. denitrificans c*NOR in the absence (black line) and presence (red line) of *cd*_1_NiR. Experimental conditions: 50 mM HEPES pH 7.0, 50 mM KCl, 0.05% DDM, 30 mM glucose, 1 U/ml glucose oxidase, 20 U/ml catalase. Once the chamber was anaerobic, cyt. c (15 µM), TMPD (0.5 mM), and 5 times 10 µM NO (from NO-saturated water) was added. At t ~250 s, ascorbate (3 mM) and cNOR (80 nM) were added. For the trace with *cd*_1_NiR (80 nM), it was added before the addition of NO. (**b**) Titration of the inhibitory effect of *cd*_1_NiR on *c*NOR catalysis. Experimental conditions as in A, except the *c*NOR concentration was 40 nM, and the *cd*_1_NiR concentration varied between 0–200 nM. *c*NOR activity (black circles) refers to the *k*_max_ at ~5 µM NO, with the *k*_max_ in the absence of *cd*_1_NiR set at 100%. Also shown is the effect of adding *cd*_1_NiR on substrate inhibition (blue circles) for NO-reduction by *c*NOR. The right y-axis refers to the [NO] where *k*_max_/2 is reached (termed *K*_i_^app^ in the text, note that this is higher than for *k*_max_).

If *c*NOR and *cd*_1_NiR interact with each other, they could influence each other’s catalytic parameters, therefore we measured the influence of the presence of *cd*_1_NiR on NO-reduction by *c*NOR (which is straightforward to measure). Surprisingly, we observed clear *inhibition* of *c*NOR-catalyzed NO-reduction in the presence of *cd*_1_NiR, see Fig. 2a. NO-reduction by the *P. denitrifi*cans *c*NOR exhibits a sigmoidal curve, due to substrate inhibition^26,27^ at NO > 10 µM. The value we report for *c*NOR activity is the maximum activity (*k*_max_, note that this *k*_max_ is not a *k*_cat_ since there is substrate inhibition at higher [NO]) observed at ~5 µM NO. In the presence of *cd*_1_NiR (Fig. 2a), two effects are observed; the maximum activity is lowered and the substrate inhibition pattern changes, see below.

We investigated the inhibitory effect as a function of *cd*_1_NiR concentration added, the raw data is shown in Supporting Fig. 1, and a plot of the maximum rate as a function of added *cd*_1_NiR is shown in Fig. 2b (and Supporting Fig. 2). The maximum activity of *c*NOR decreases gradually the more *cd*_1_NiR is added and the effect reaches a maximum level of inhibition (~50%) at ~30 nM *cd*_1_NiR (approximately equimolar to *c*NOR). Surprisingly, at higher concentrations of *cd*_1_NiR, the inhibition is released (Fig. 2b), the possible reasons for this are discussed further below (see Fluorescence section). For the investigations of the influence of the electron donor described in the next section, we used the *cd*_1_NiR concentration (and *c*NOR/*cd*_1_NiR ratio) giving the maximum inhibition.

### Electron donation to *c*NOR in the presence of *cd*_1_NiR

In the co-crystal structure of the complex between the *cd*_1_NiR and *c*NOR from *P. aeruginosa*^24^, the interaction surface (see Fig. 1) could possibly overlap with interaction of the electron donor to *c*NOR. Thus, one reason for the inhibition observed with *cd*_1_NiR could be that it interferes with electron donation, and we therefore studied the titration behavior of electron donors for *c*NOR catalysis in the absence and presence of *cd*_1_NiR.

**Figure 3:**
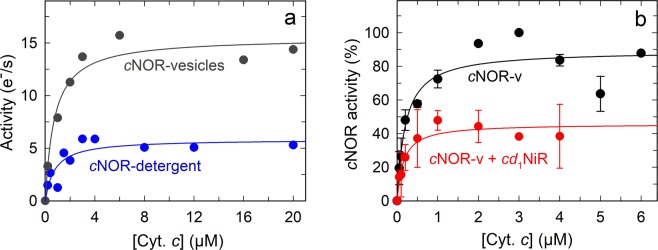
Determinations of the *K*_m_ for cytochrome *c* for NO reduction by *c*NOR. **(a**) Comparison between *c*NOR in detergent (blue) and reconstituted in liposomes (grey). Experimental conditions as in Fig. 2, except for with liposomes, DDM was omitted. *c*NOR activity refers to the *k*_max_ at ~5 µM NO with the *k*_max_ obtained without cyt. *c* subtracted. The lines shown are fits giving *k*_cat_ = 6 ± 1 (e^-^/(s•*c*NOR)), *K*_m_ = 0.8 ± 0.3 µM cyt. *c* (blue, detergent) and *k*_cat_ = 15 ± 1 (e^-^/(s•*c*NOR)), *K*_m_ = 0.8 ± 0.2 µM cyt. *c* (dark grey, liposomes). (**b**) Comparison between liposome-reconstituted *c*NOR in the absence (black) and presence (red) of *cd*_1_NiR. *c*NOR activity refers to the *k*_max_ at ~5 µM NO, with the *k*_max_ at 3 µM cyt. *c* in the absence of *cd*_1_NiR set at 100%. The curves were fitted as in a, giving *K*_m _ = 0.20 ± 0.05 µM cyt. *c* (black, *c*NOR only) and *k*_cat_ = 46 ± 4%, *K*_m_ = 0.15 ± 0.05 µM cyt. *c* (red, + *cd*_1_NiR). Experimental conditions as in **(a**).

As a pre-requisite for the investigation of possible interference of electron donation caused by *cd*_1_NiR binding to *c*NOR, we determined the *K*_m_ for cytochrome *c* (horse heart) during NO reduction by *c*NOR. For these titrations, we always used the maximum activity, *k*_max_ at ~5 µM NO. The results are shown in Fig. 3a and can be fitted with a *k*_max_ = 6 ± 1 e^−^s^−1^ (electrons/(s•*c*NOR)) and *K*_m_ = 0.8 ± 0.3 µM. As seen in this graph, the data is scattered and the standard deviation in the *K*_m_ quite large. We therefore instead measured the activity with *c*NOR reconstituted into liposomes. The aim of this was two-fold, first the activity of *P. denitrificans c*NOR is higher in liposomes^17,28^, giving us a larger total change in activity during titration and hence smaller relative errors. Secondly, the presence of a membrane might influence a putative *c*NOR-*cd*_1_NiR interaction, as suggested for the *P. aeruginosa* complex^24^. In liposome-reconstituted *c*NOR, we determined the *k*_max_ to 15 ± 1 e^−^s^−1^ and the *K*_m_ for cyt. *c* to 0.8 ± 0.2 µM (Fig. 3a), i.e. no change in *K*_m_ was observed.

Side by side experiments were conducted to determine the *K*_m_ for cyt. *c* of liposome-reconstituted *c*NOR in the absence or presence of ∼30 nM *cd*_1_NiR (Fig. 3b). This is the *cd*_1_NiR concentration which maximally inhibits detergent-solubilized *c*NOR (Fig. 2b; see also corresponding data for liposome-reconstituted *c*NOR in Supporting Fig. 2). Surprisingly the observed *K*_m_ was unchanged in the presence of *cd*_1_NiR (*K*_m_ = 0.15 ± 0.05 µM) compared to the control (*K*_m_ = 0.20 ± 0.05 µM) as shown in Fig. 3b. However, the relative *k*_max_ in the presence of *cd*_1_NiR was ~50% of the control. Thus, only the *k*_max_ and not the *K*_m_ value is affected, indicating that the *cd*_1_NiR and cyt. *c* do not bind at the same place to *c*NOR.

We note that the *K*_m_ value determined (in the absence of *cd*_1_NiR) in this experiment is different from that determined in the previous experiment (Fig. 3a). This is probably due to the *K*_m_ values being low and therefore the data obtained possibly not represented well by a simple Michaelis-Menten fit. Also, the concentration of *cd*_1_NiR is about equimolar to *c*NOR and small differences in the relative concentrations between experiments might affect the data. These considerations are the reasons for doing comparative experiments ‘side-by-side’.

Since the *K*_m_ for cyt. *c* does not change significantly in the presence of *cd*_1_NiR, we scrutinized the raw data used for Fig. 3b, and re-plotted it without subtracting the background rate (with Ascorbate (Asc)/tetramethyl-p-phenylenediamine (TMPD)) (see Supporting Fig. 3A). This shows that there is inhibition of the basal activity by *cd*_1_NiR with only Asc/TMPD to provide electrons that does not change significantly when cyt. *c* is added. This observation suggests that *cd*_1_NiR inhibits the electron donation from TMPD rather than that from cyt. *c*. To verify this, we studied the effect of titrating *cd*_1_NiR on *c*NOR catalysis in the absence of TMPD (with only cyt. *c* and Asc), see Supporting Fig. 3B which shows that in the absence of TMPD, there is no inhibition.

**Figure 4:**
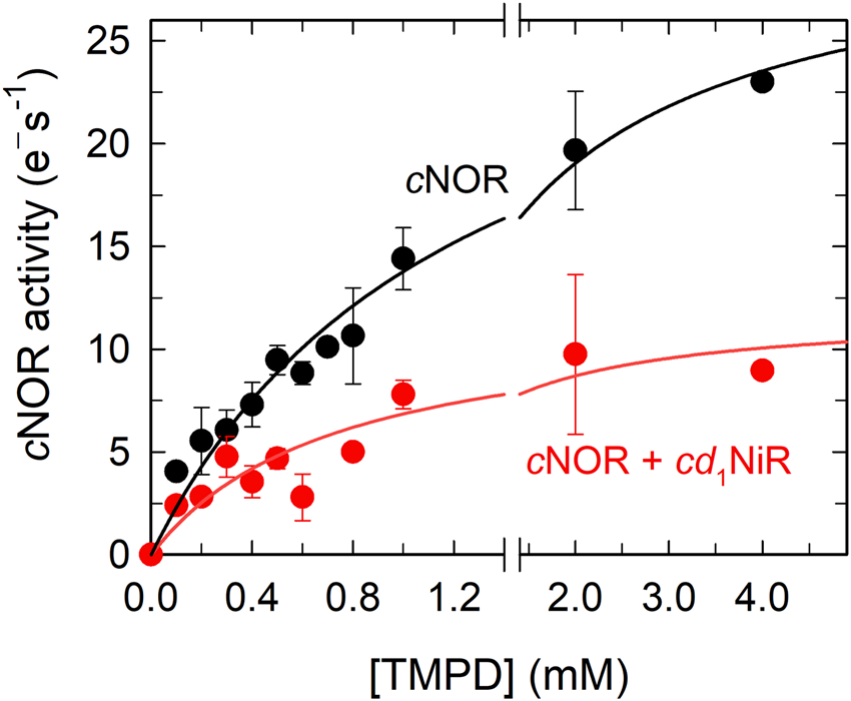
Determination of the *K*_m_ for TMPD for NO reduction by liposome-reconstituted *c*NOR in the absence (black) and presence (red) of *cd*_1_NiR. Experimental conditions, and data treated as in Fig. 3. The black line is a single-hyperbolic fit to the *c*NOR data giving *k*_cat_ = 31 ± 3 (e^−^/(s•*c*NOR)), *K*_m_ = 1.2 ± 0.2 mM TMPD. The red line is the same fit for the *c*NOR + *cd*_1_NiR data, giving *k*_cat_ = 10 ± 2 (e^−^/(s•*c*NOR)), *K*_m_ = 0.6 ± 0.2 mM TMPD.

We then studied the *c*NOR activity as a function of the TMPD concentration (with ascorbate, but in the absence of cyt. *c*), both in the absence and presence of *cd*_1_NiR, see Fig. 4. Here there is a clear inhibition by *cd*_1_NiR. The data indicates that there might be more than one interaction with TMPD, but assuming a single binding site, the obtained constants are; in the absence of *cd*_1_NiR: *k*_max_ = 31 ± 2 e^−^s^−1^ and *K*_m_ = 1.2 ± 0.2 mM, and in the presence of *cd*_1_NiR: *k*_max_ = 12 ± 2 e^−^s^−1^ and *K*_m_ = 0.7 ± 0.2 mM. In this scenario, both the *k*_max_ and *K*_m_ are affected (so-called mixed inhibition). Our data does not allow for any unambigous fit to a more complex behaviour.

We also observe inhibition by *cd*_1_NiR when PMS is used as an electron mediator instead of TMPD (see Supporting Fig. 4). Thus, there is an inhibition of *c*NOR activity by the presence of *cd*_1_NiR with both TMPD and PMS, indicating that the interaction surface (or part of it, cf. the data with TMPD) on *c*NOR is similar for TMPD and PMS, and that this surface overlaps with *cd*_1_NiR binding.

As controls for the measurements described above, we studied the possibility that TMPD directly affects auto-reduction of NO, as well as the possibility that small amounts of nitrite formed (from NO) in the buffer could have effects interfering with our results. However, we found no effects that were significant enough to influence the data presented. For nitrite, we see that it can inhibit *c*NOR activity, but only at high (mM) concentrations, consistent with previous studies^29^.

### Substrate inhibition in *c*NOR in the presence of *cd*_1_NiR

As described above, adding *cd*_1_NiR during NO-reduction by *c*NOR has two effects; both reducing the maximum activity investigated above, and in changing the pattern of substrate inhibition, see Fig. 2 and Fig. S1. Thus, a plot of the NO concentration where *k*_*max*_/2 is reached as a function of *cd*_1_NiR added is shown in Fig. 2b (together with the corresponding effects on the *k*_max_). Note that this refers to the NO concentration at *higher* NO (than that which gives *k*_max_) where *k*_max_/2 is reached, and therefore refers to an apparent *K*_i_ (rather than an apparent *K*_m_). We note that the decrease in maximum rate at low *cd*_1_NiR correlates well to the decrease in the *K*_i_^app^ for NO (that is a higher apparent affinity for NO at an inhibitory site), whereas the *K*_i_^app^ for NO then roughly saturates at ~40 nM *cd*_1_NiR. It is thus clear that even though the inhibition on the maximum rate is released at higher *cd*_1_NiR, there is still an influence also at higher *cd*_1_NiR concentrations, indicating an interaction between *c*NOR and *cd*_1_NiR that persists (see Discussion).

### SDS page analysis for localization of *cd*_1_NiR in *P. denitrificans*

To investigate the localization of *cd*_1_NiR in *P. denitrificans* cells grown under denitrifying conditions, cells were fractionated, and the presence of *cd*_1_NiR analyzed using Western blot with a specific antibody for *cd*_1_NiR. The results, shown in Supporting Fig. 5, demonstrate that although *cd*_1_NiR is present mainly in the periplasm, it is also found in the membrane fraction. We investigated many different conditions for this analysis including different detergents and ionic strength, but although using a milder detergent (digitonin) for solubilisation of the membrane fraction gave a somewhat larger fraction of *cd*_1_NiR bound to it, this fraction is still small, see Discussion.

**Figure 5:**
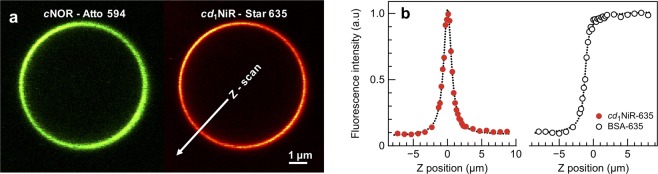
Fluorescently labeled *c*NOR (ATTO 594) and *cd*_1_NiR (STAR 635) visualized with a laser scanning confocal microscope. (**a**) *c*NOR was reconstituted in giant unilamellar vesicles (GUVs) and *cd*_1_NiR was added to the GUV solution. *c*NOR was detected in the membrane (green GUV) and *cd*_1_NiR was found to be highly associated with the membrane (red GUV). (**b**) Fluorescence intensity scan across the membrane (Z-plane), from the inside (−) to the outside (+) of a GUV after adding *cd*_1_NiR or BSA, labeled with STAR 635. *cd*_1_NiR (in contrast to BSA) is highly enriched at the membrane surface. For experimental conditions, see Material and Methods.

### Interactions of *cd*_1_NIR and *c*NOR investigated by fluorescence spectroscopy

Purified *cd*_1_NiR and *c*NOR proteins were fluorescently labeled with ATTO 594 and STAR 635, respectively. *c*NOR was successfully reconstituted in giant unilamellar vesicles (GUVs), anchored to a biotin-covered glass surface imaged using a confocal laser scanning microscope (see Fig. 5a, green GUVs). *cd*_1_NiR was then added in equimolar concentration (to *c*NOR) to the GUV solution. The *cd*_1_NiR was also added to ‘empty’ GUVs. The *cd*_1_NiR was found to be highly associated with the membrane (Fig. 5a, red GUV). A scan across the membrane (Z-plane, Fig. 5b) showed an increase in fluorescence intensity in the membrane plane (Z = 0), i.e. the concentration of *cd*_1_NiR is higher at the membrane surface than in the surrounding solution. A control with STAR 635-labeled BSA protein (Fig. 5b) showed no such increase in the membrane plane.

The possible interaction between *cd*_1_NiR and *c*NOR was then assayed using fluorescence correlation spectroscopy (FCS, see Methods) in combination with the confocal setup. First, we measured FCS on the reconstituted *c*NOR-ATTO 594 and *cd*_1_NiR-STAR 635 simultaneously on the membrane surface and looked for interaction by using two-color FCS and cross-correlation analysis (FCCS). However, no significant interaction could be distinguished by this approach (Supporting Fig. 6). In agreement with this, there was no significant difference in the degree of *cd*_1_NiR binding between the GUVs with or without *c*NOR reconstituted.

**Figure 6:**
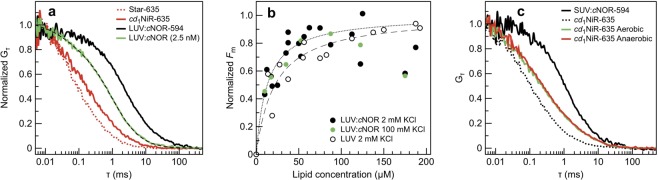
Interactions between *cd*_1_NiR and liposomes, *c*NOR-liposomes and native membranes. (**a**) Fluorescence autocorrelation curves measured on a sample containing 5 nM *cd*_1_NiR-STAR 635 (red), and after addition of LUVs containing 2.5 nM *c*NOR (green). The dashed (black) line is a fit of the data using a model with two diffusion times. As references, measurements of a sample containing free dye STAR 635 (red dotted line) and a sample with LUVs containing *c*NOR labelled with ATTO 594 are also shown. (**b**) Titration of 5 nM *cd*_1_NiR-STAR 635 with increasing concentrations of LUVs containing unlabeled *c*NOR in buffer containing 2 mM KCl (black) or 100 mM KCl (green), and titration with the same amount of LUVs without protein (white). The plot shows the amplitude of the slow component where F_max_ has been set to 1. The data for LUVs with and without protein were fitted with a simple binding model (see text for details). (**c**) FCS curves from titration experiments with native membranes from *P. denitrificans* grown under aerobic (green) or anaerobic denitrifying (red) conditions. Sonicated membranes were added to a solution of 50 nM *cd*_1_NiR-STAR 635 (black dotted line). A sample with DOPC-liposomes produced in the same way containing *c*NOR labelled with ATTO 594 is shown as reference (black line).

To further probe potential interactions, *c*NOR was reconstituted in DOPC liposomes (LUVs) and the interaction with *cd*_1_NiR was assayed by monitoring changes in diffusion of labelled *cd*_1_NiR upon binding. The diffusion time of the liposomes containing *c*NOR labelled with ATTO 594 was 2.6 ms determined with FCS (Fig. 6b, black trace). This would correspond to a liposome size of approximately 90 nm (corresponding well to the 100 nm expected from the LUV-forming protocol). Liposomes containing unlabeled *c*NOR were added in increasing concentrations to a solution containing 5 nM *cd*_1_NiR-STAR 635. The diffusion of *cd*_1_NiR-STAR 635 alone was 0.34 ms, corresponding to a hydrodynamic radius of ~100 Å. The addition of liposomes changed the apparent diffusion time of *cd*_1_NiR indicating that *cd*_1_NiR binds to the LUVs containing *c*NOR (Fig. 6). The fraction of bound *cd*_1_NiR was determined by fitting the FCS data with a two-component diffusion model (Eq. 1), where the amplitude of the component with a long (2.6 ms) diffusion time was taken to represent liposome-bound *cd*_1_NiR. The amplitudes of the 2.6 ms component (Fig. 6b) were fitted with a simple ligand-binding model (Eq. 2). The same experiment was repeated with ‘empty’ LUVs, and the binding constant for liposome binding to *cd*_1_NiR, compared on the basis of lipid concentration, was ~13 ± 1 μM with and ~22 ± 2 μM (see Fig. 6b) without *c*NOR present in the membrane. This difference is likely within the experimental uncertainties and *cd*_1_NiR has a similar, rather high, affinity to the liposomes independently of the presence or absence of *c*NOR.

We also increased the ionic strength in the buffer from 2 to 100 mM (KCl) in order to shield purely electrostatic interactions between *cd*_1_NiR and the membrane. However, no decrease in the fraction bound *cd*_1_NiR was observed (green circles in Fig. 6b) which indicates that the association of *cd*_1_NiR to the DOPC membrane is not purely electrostatic in nature (see Discussion).

Although diffusion of both *cd*_1_NiR and *c*NOR was detected when measuring FCS on the GUV membrane surface, there was no interaction observed using cross-correlation analysis. Thus, neither the titration experiment using small liposomes, nor the FCCS, measured directly on the membrane surface, could detect an interaction between *cd*_1_NiR and *c*NOR going beyond the interaction between *cd*_1_NiR and the membrane under these experimental conditions. However, it should be noted that the rather high-affinity interaction between *cd*_1_NiR and the DOPC liposomes themselves might ‘hide’ a relatively weak interaction between *c*NOR and *cd*_1_NiR, see Discussion.

Since *cd*_1_NiR showed such significant interaction with the pure DOPC liposomes, we also wanted to investigate whether an interaction between *cd*_1_NiR and the membrane (with or without *c*NOR expressed) could be observed using native *P. denitrificans* membranes. Small membrane vesicles were made from cells grown under either aerobic or anaerobic denitrifying conditions and mixed with a solution containing 50 nM *cd*_1_NiR-STAR 635. Although *c*NOR expresses only during denitrifying conditions no differences were observed. The diffusion time of *cd*_1_NiR-STAR 635 (Fig. 6c) was partly slowed down in both cases with a fraction matching the diffusion time (~1.5 ms) of sonicated DOPC liposomes containing *c*NOR-ATTO 594. In both cases the slow fraction was maximum ~ 25% of the total *cd*_1_NiR-STAR 635 population, in comparison to up to 85% when using pure DOPC liposomes. Although these fractions do not necessarily correspond directly to the fraction bound *cd*_1_NiR, we can conclude that *cd*_1_NiR interacts much more strongly with artificial ‘lipid-only’ liposomes than it does with native membranes, see Discussion.

### Dimerization of *cd*_1_NiR

**Figure 7:**
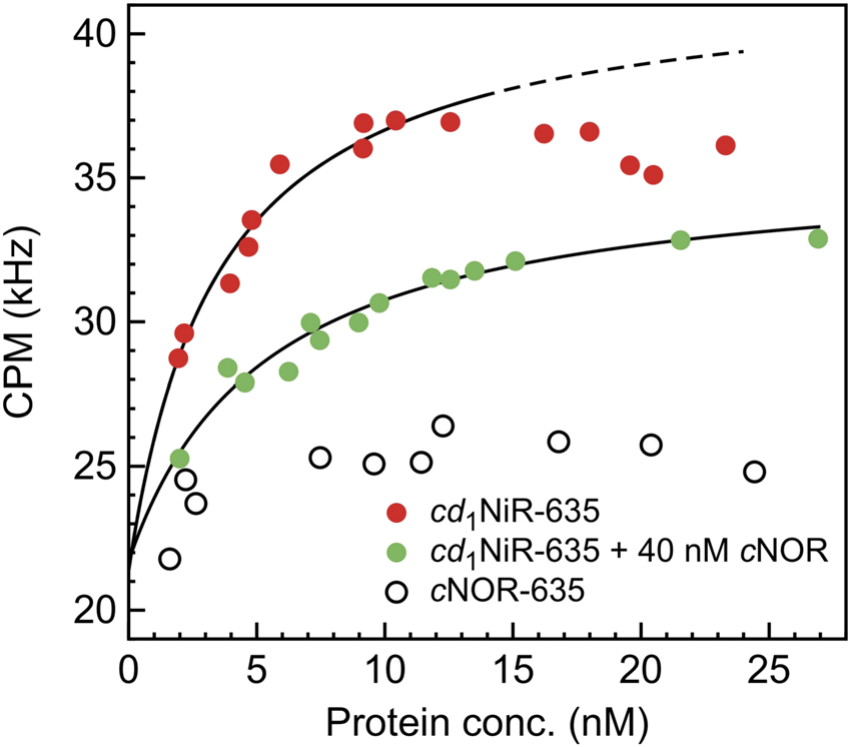
Fluorescence intensity (count per molecule (CPM)) when adding increasing amounts of *cd*_1_NIR-STAR 635 to a solution with (green circles) or without (red circles) 40 nM *c*NOR. The fluorescence intensity measured with increasing concentrations of *c*NOR-STAR 635 alone is also shown (white circles). The black lines are ligand-binding fits for *cd*_1_NiR dimerization, giving K_D_ = 3.5 ± 0.1 nM (without) and 5.3 ± 0.2 nM (with *c*NOR) respectively.

From the functional *c*NOR-catalyzed NO-reduction data presented above, the observed effect of adding increasing amounts of *cd*_1_NiR (see Fig. 2b) made us consider that this dependence could be linked to dimerization of *cd*_1_NiR. *cd*_1_NiR is a dimer in the X-ray crystal structures from both *P. aeruginosa*^9,24^ and *P. denitrificans*^6^ and also reported to be a dimer in solution^5,30^, but to our knowledge, there is no reported value for the dimerization constant. To further investigate if there is such a *cd*_1_NiR dimer dissociation/association in the concentration range used, we analyzed the fluorescence intensity from labeled *cd*_1_NiR as a function of its concentration. The fluorescence intensity as well as the particle number of *cd*_1_NiR-STAR 635 obtained by FCS (parameter *N* in Eq. 1) was used to determine the photon count-rate per molecule (CPM). Figure 7 shows that the CPM increases with increasing concentrations when adding *cd*_1_NiR-STAR 635 alone. Assuming that the majority of *cd*_1_NiR-STAR 635 is present as a monomer at very low concentrations (<1 nM) an increase in CPM at higher concentrations indicates dimerization. In comparison, the CPM of *c*NOR labelled with the same fluorophore (*c*NOR-STAR 635) showed only a small increase, indicating that there is no change in its oligomeric state in this region.

This *cd*_1_NiR titration was done both in the presence and absence of *c*NOR. Interestingly, both the maximum CPM for *cd*_1_NiR-STAR 635 and its concentration dependence changed when 40 nM *c*NOR (unlabeled) was present in the solution. The CPM data could be fitted using the ligand-binding model (Eq. 2) allowing for a simple comparison; the apparent binding constants for the suggested dimerization of *cd*_1_NiR-STAR 635 was 3.5 ± 0.1 nM without and 5.3 ± 0.2 nM with *c*NOR present. We observed a slight decrease in CPM at *cd*_1_NiR-STAR 635 concentrations above 15 nM, but only in the case when *c*NOR was not present and these data points were not included in the fit (dashed line). The maximum CPM reached was lower in the presence of *c*NOR, indicating either a quenching effect, or that even when dimerized, the *cd*_1_NiR is influenced by/binds *c*NOR, or that there is a fraction of *cd*_1_NiR that cannot dimerize in the presence of *c*NOR.

## Discussion

The denitrification process is tightly controlled in *P. denitrificans*, in order to avoid release and accumulation of toxic intermediates; nitric oxide and (to a lesser degree) nitrite. This control occurs on the level of transcription, by a tight coupling of the expression of the enzymes involved (see e.g.^31,32^). It has also been suggested that *in vivo*, kinetic parameters for *c*NOR are significantly different from those obtained *in vitro*, with e.g. a very high NO affinity thereby helping to keep the steady-state NO levels low^3^. A different way to minimize toxic intermediates would be to control the enzymes themselves, by e.g. forming a functional complex between *cd*_1_NiR and *c*NOR that shuttles the NO produced from *cd*_1_NiR directly to *c*NOR without release into the bulk phase. Support for this hypothesis was recently presented in the form of a co-complex structure of the *cd*_1_NiR and *c*NOR from *P. aeruginosa* obtained from separately purified components^24^, see Fig. 1. It should be noted that in aerobic respiration in both eukaryotes and prokaryotes, supercomplexes of individual enzyme components involved are frequently found (see e.g.^22,23,33^ and references therein).

In this work, we investigated the possibility of a *c*NOR/*cd*_1_NiR complex for *P. denitrificans*, a denitrification model bacterium. The two enzymes *cd*_1_NiR and *c*NOR from *P. denitrificans* share 48% (*c*NOR) and 61% (*cd*_1_NiR) overall sequence identity with their counterparts from *P. aeruginosa*. We purified the *cd*_1_NiR from *Paracoccus pantotrophus* and not *denitrificans*, but these two enzymes are 97% identical.

The addition of *cd*_1_NiR during NO-reduction by *c*NOR shows some intriguing effects. First, both the substrate inhibition pattern and maximum rate of NO reduction is affected by *cd*_1_NiR (Fig. 2). Since both these parameters are presumed to be linked to the effective electron donation (see ref.^34^ for a discussion on substrate inhibition), it seems plausible that a complex of *cd*_1_NiR and *c*NOR forms and that the complex interface interferes with the access of the electron donor to *c*NOR. This conclusion is supported by the co-crystallised *Ps. aeruginosa cd*_1_NiR/*c*NOR complex^24^, which shows that the *cd*_1_NiR interacts with the NorC subunit (see Fig. 1) that harbors the initial electron acceptor (a heme *c*) of *c*NOR. As is clear from Fig. 3b (and Supporting Fig. 3) however, the observed *K*_m_ for cyt. *c* does not change in the presence of *cd*_1_NiR but direct electron donation by TMPD is clearly affected (see Fig. 4). Although we have not used the presumed physiological *c*^550^ cytochrome^7,35^, but the readily available horse heart (*hh*) cyt. *c*, the structures align very well and *hh* cyt. *c* works well as electron donor to *c*NOR. The small TMPD molecule (MW: 164 g/mol) presumably has a less defined or multiple interaction surfaces on *c*NOR, as indicated by our titration data (Fig. 4), and these (or some of them) presumably overlap with the interaction surface for *cd*_1_NiR. We also observe inhibition by the presence of *cd*_1_NiR with the electron mediator PMS (instead of TMPD, see Supporting Fig. 4).

An interesting parallel is that the antibody used for crystallisation of the *Ps. aeruginosa c*NOR (only)^21^, was shown to interfere with electron donation from cytochrome *c*, but not from PMS^21^. The binding site for this antibody has some, albeit small, overlap with the binding of *cd*_1_NiR in the co-crystal complex^24^.

The inhibition of *c*NOR activity observed upon addition of *cd*_1_NiR shows a clear correlation in extent to the concentration of added *cd*_1_NiR up until approximately equimolar amounts to *c*NOR (20–40 nM), but at higher concentrations of *cd*_1_NiR, the inhibition is relieved. This is a surprising but highly reproducible observation which we suggest could be due to an effect of dimerization of *cd*_1_NiR, which is purified and crystallized in the dimeric form both in *P. pantotrophus*^6^ and *P. aeruginosa*^9^. Our fluorescence intensity measurements with labeled *cd*_1_NiR showed a fluorescence ‘count-rate per particle’ (CPM) increase (Fig. 7**)**, consistent with dimerization with an apparent *K*_D_ of ~3.5 nM. To our knowledge, an apparent dimerization constant for *cd*_1_NiR has not previously been determined. In the *P. aeruginosa cd*_1_NiR dimer there is domain ‘swapping’ between the monomers, leading to a presumably obligatory dimer, whereas no such swapping occurs in the *P. pantotrophus* (and hence *denitrificans) cd*_1_NiR. This difference is likely to affect the stability of the dimer and also the propensity to interact with *c*NOR. Also consistent with our functional data is that this *K*_D_ is affected (increases) in the presence of *c*NOR, from 3.5 nM to ~5 nM, supporting an interaction between *c*NOR and *cd*_1_NiR in the same concentration range as used in the functional assay. The total CPM for *cd*_1_NiR is also affected by *c*NOR, and the 40 nM *c*NOR used in this experiment might not be enough to saturate the effects, such that the influence of *c*NOR for *cd*_1_NiR dimerization might be somewhat underestimated.

The *Ps. aeruginosa* co-complex structure, where each *cd*_1_NiR monomer binds a *c*NOR on opposite ‘ends’ (Fig. 1) is a structure that cannot be formed *in vivo* because of the restrictions imposed by the cytoplasmic membrane. This is thus consistent with a *c*NOR/*cd*_1_NiR interaction that is stronger when both proteins are in their monomeric forms. In this context, we do see differences in the inhibition patterns when adding *cd*_1_NiR to *c*NOR in detergent versus in liposomes, but qualitatively, the results are similar (see Supporting Fig. 2).

Although the effect *cd*_1_NiR has on the maximum rate of NO-reduction was interpreted above in terms of only occurring for the monomer of *cd*_1_NiR, even at higher *cd*_1_NiR concentrations (i.e. when *cd*_1_NiR is predominantly a dimer) it still influences *c*NOR catalysis as seen in the plots of the *K*_i_^app^ (Fig. 2b). A possible interpretation for this is that *cd*_1_NiR still interacts with *c*NOR even in its dimeric form, but that the interaction surface changes. It’s also possible that the effect on the *c*NOR substrate inhibition pattern originates from structural changes occurring in *cd*_1_NiR itself as a response to changes in [NO] or in reduction levels (as seen in^36^, see below).

Even though there are clear influences on the function of *c*NOR by the presence of *cd*_1_NiR, we could not find evidence for a high-affinity, constant complex between the *P. denitrifi*cans *cd*_1_NiR and *c*NOR, as indicated e.g. by the Western Blot results (Supporting Fig. 5) and the lack of clear differences between the interactions of (fluorescently labeled) *cd*_1_NiR with either the native aerobic or anaerobic (denitrifying) *P. denitrifi*cans membranes shown in Fig. 6c. Interpreting this data is complicated by the observation that there is a rather high affinity of the *cd*_1_NiR for lipid membranes (see Figs. 5 and 6), as reported also previously^37,38^. This interaction is not purely electrostatic in nature, whereas only electrostatic interactions between the *d*_1_ domain and the membrane were discussed for the *P. aeruginosa cd*_1_NiR-*c*NOR co-complex simulations^24^. An interaction between *cd*_1_NiR and the cytoplasmic membrane would enable the NO produced to directly dissolve into the membrane bilayer from which it can migrate to the gas channel in *c*NOR (see Fig. 1) without equilibrating with the bulk water phase even with no direct contact between the two enzymes.

**Figure 8:**
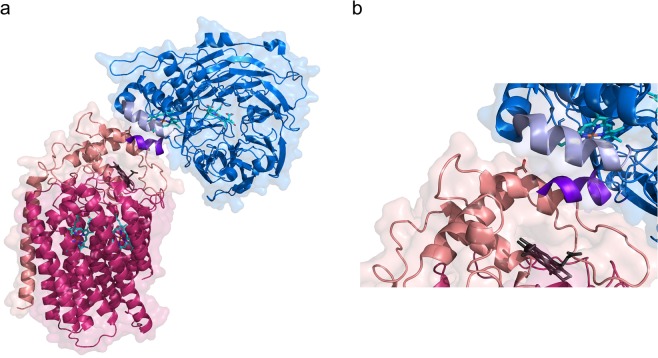
(**a**) The model of the *P. denitrificans c*NOR (NorB in magenta, NorC in salmon) and the *P. pantotrophus cd*_1_NiR monomer structure (blue, PDB ID: 1QKS^6^) overlapped on the *cd*_1_NiR/*c*NOR co-complex structure from *P. aeruginosa* (PDB ID: 5GUW^24^ (not shown)). The *cd*_1_NiR helix that interacts with NorC (in the co-complex) in light blue and the ‘extra’ N-terminal helix in purple. Note that this ‘extra’ helix would clash into the NOR/NiR interface. Shown is also the D123 (stick) of NorC (equivalent to the E-119 in *Ps. aeruginosa*) and the heme groups of the proteins. (**b**) Zoom-in of interface. The picture outlines also the surface (transparent) of the proteins except for the ‘extra’ helix.

Since a co-complex structure of *cd*_1_NiR-*c*NOR exists only for the *P. aeruginosa* proteins, we overlayed the potential interaction between the two homologous proteins from *P. denitrifi*cans. For the *P. denitrificans c*NOR, we constructed a model based on the *P. aeruginosa* structure (to which it has 54% (NorB) and 47% (NorC) sequence identity), as shown in Supporting Fig. 8A, and the Glu-119 of *P. aeruginosa c*NOR that forms the main interaction with the *P. aeruginosa cd*_1_NiR overlays well with the corresponding Asp-123 in *P. denitrifi*cans *c*NOR. However, the *P. pantotrophus cd*_1_NiR structure (sequence identity 97% to *P. denitrifi*cans *cd*_1_NiR) shows significant differences to the *cd*_1_NiR from *P. aeruginosa*, and there is no Arg equivalent to the R-96 (numbering from our alignment (Supporting Fig. 7), corresponds to the R-71 in the alignment from Terasaka *et al*.^24^) that interacts with the E-119 on *c*NOR (in *P. denitrificans and P. pantotrophus cd*_1_NiR, the corresponding residue is a Leu). The structural overlay of the *P. pantotrophus* and *P. aeruginosa cd*_1_NiRs (see Supporting Fig. 8B) further shows that the cyt. *c* domain is more different than the *d*_1_ domain and specifically the region on *cd*_1_NiR that is interacting with *c*NOR in the *Ps. aeruginosa* co-crystal structure is markedly different in *P. pantotrophus cd*_1_NiR, there is a small N-terminal helix that would ‘clash’ with the *c*NOR, as shown in Fig. 8, whereas in *Ps. aeruginosa cd*_1_NiR, the N-terminal is involved in ‘domain swapping’ and forms part of the *d*_1_ domain (see Supporting Fig. 8C).

However, it is also known that the *P. pantotrophus cd*_1_NiR *c* domain structure is significantly different in the reduced state^36^, and thus suggested to undergo large-scale conformational changes upon reduction (Supporting Fig. 9). Such changes could affect both the *c*NOR interaction and the dimerization constant, since the *c* domain of *cd*_1_NiR ‘swings’ out of dimer contact in the reduced state. It is difficult to predict what would happen to a putative *cd*_1_NiR/*c*NOR complex when *cd*_1_NiR is reduced since the N-terminal ‘clashing’ helix **(**Fig. 8**)**, is not even resolved in the reduced *cd*_1_NiR structure^36^, and there is hardly any overlap between the oxidised and reduced *c* domain structures (Supporting Fig. 9). Presumably the interaction between *P. denitrificans c*NOR and *cd*_1_NiR, if it occurs using a similar interaction surface as in *Ps. aeruginosa*, would affect this *cd*_1_NiR conformational change and hence could be involved in controlling *cd*_1_NiR activity. We also note that in our functional *c*NOR assays, *cd*_1_NiR is presumably predominantly in the reduced state (depending on if it has turned over, see^39^) since there is an excess reductant and no nitrite added.

So, are there physiological consequences of having an interaction between the *cd*_1_NiR monomer and *c*NOR that becomes much less pronounced once the *cd*_1_NiR dimerizes? It is possible that such regulation on the enzyme level (on top of the major transcriptional regulation) is there to fine tune flux through denitrification in response to rapidly fluctuating environmental conditions and is especially important when expression levels are low.

## Materials and Methods

### *cd*_1_NiR; growth of bacteria and purification

*Paracoccus pantotrophus* (G6) was grown anaerobically and *cd*_1_NiR purified essentially as in^5^. Briefly, bacteria were grown until OD_600_ ~0.8 in a medium containing nitrate as electron acceptor and acetate as carbon source, supplemented with 50 µg/ml kanamycin. To obtain the periplasmic fraction, the cell pellet was resuspended in 200 mL buffer containing 0.5 M sucrose, 3 mM EDTA, 100 mM Tris-HCl, pH 8.0, and 400 mg of lysozyme was added. The solution was then incubated with constant stirring at 30 °C for 40 minutes. The cell solution was then centrifuged at 25000 g for 10 min. The supernatant (containing the periplasm) was applied to a DEAE anionic-exchange column (GE Healthcare), from which bound fractions were eluted with a 0–300 mM NaCl gradient in 100 mM Tris/HCl pH 8.0. The brown-colored fractions were pooled, solid ammonium sulfate was added to 40% (w/v) and the precipitated protein removed by centrifugation at 30000 g for 30 min.

The solution was applied to a phenyl-sepharose column (GE Healthcare), and a 40-0% ammonium sulfate gradient was applied. The fractions that contained pure *cd*_1_NiR (A^406^/A^280^ ~1.25)^8^ were pooled and concentrated. The concentration of *cd*_1_NiR was determined by using ε^418^ = 268 mM^−1^ cm^−1^. For antibody generation the enzyme was further purified using size exclusion chromatography in 100 mM Tris/HCl pH 7.0 on a Superose 10/300 column (GE-Healthcare).

### *c*NOR; growth of bacteria, protein purification and model building

Purification of *c*NOR (*P. denitrificans* overexpressed in *E. coli*) was performed as described in^18^, based on the original protocol from^40^. Briefly, the plasmid pNOREX was transformed in to a JM109 strain which contained the pEC86 vector^40^. *c*NOR expression was induced by IPTG. The membranes were solubilized in 100 mM Tris, pH 7.6, 50 mM NaCl, 1 mM EDTA and 1% n-dodecyl-β-D-maltoside (DDM). The membrane solution was incubated with constant stirring for 1 hour at 4 °C. The unsolubilized membranes were removed by centrifugation, and the supernatant was applied to a Q-Sepharose high performance (GE-HealthCare) column, which was equilibrated in 20 mM Tris/HCl pH 7.6, 0.04% DDM and 5 mM NaCl. The column was washed with the same buffer but containing 250 mM NaCl and *c*NOR was eluted with a 250 mM-500 mM NaCl gradient in 20 mM Tris/HCl pH 7.6, 0.04% DDM.

The pure fractions of *c*NOR were pooled, diluted 3 times in 100 mM Tris/HCl, 50 mM NaCl, and the concentration of NaCl was lowered to below 50 mM by repeated dilution and reconcentration in concentrating vials (Millipore Merck, Ltd). Aliquots were flash frozen in liquid nitrogen and stored in −80 °C.

The structural model of *P. denitrifi*cans *c*NOR was constructed with SWISS-MODEL (https://swissmodel.expasy.org) using the default parameters and refinement procedure. The crystal structure of *Pseudomonas aeruginosa c*NOR (PDB ID: 3o0r^21^, sequence identity 54% for NorB and 47% for NorC) was used as the structural template. The *P. denitrifi*cans *c*NOR could also be modelled on the *Roseobacter denitrificans c*NOR (sequence identity 75% for NorB and 69% for NorC) structure^41^, but since this *c*NOR, unlike *P. denitrifi*cans *c*NOR, was found to bind a Cu^+^ ion in the NorC subunit, which could potentially influence the region around the presumed interaction with *cd*_1_NiR, we chose to use the *P. aeruginosa c*NOR–derived model.

### Detection of *cd*_1_NiR in anaerobically grown (on nitrate) *P. denitrificans* cells

*P. denitrificans* (Pd1222) cells were grown anaerobically on nitrate (32 mM) as electron acceptor at 37 °C, the cells were harvested and the periplasm was obtained by osmotic shock as described above. The pellet was sonicated, and the membranes were extracted by high-speed centrifugation (100 000 g). The different cell components (whole cell, periplasm (PL) and membrane (M) fractions) were subjected to SDS-PAGE (Invitrogen, 4–12%) analysis followed by Western blot using a PDVF membrane and an antibody against *cd*_1_NiR, obtained from Biogenes GmbH (Germany).

### Protein reconstitution in vesicles

For the generation of small unilamellar vesicles (SUVs), a solution of 40 mg/ml soybean lipids in 50 mM Tris/HCl pH 7.0, 50 mM KCl was sonicated until it became clear. 2–4 µM *c*NOR was added to the liposomes in the presence of 0.6% Na-cholate and the mixture was incubated for 1 hour at 22 °C. The detergent was then removed on a PD-10 column (GE-Healthcare). For generation of large unilamellar vesicles (LUVs), DOPC (1,2-dioleoyl-sn-glycero-3-phosphocholine) lipids dissolved in CHCl_3_ were dried and then rehydrated to 2 mM in a 10 mM phosphate buffer (pH 7.4) with 2 mM KCl. Unilammelar liposomes were made by passing the lipid solution trough a filter with a 100 nm pore size 21 times. *c*NOR was reconstituted into the liposomes by gently solubilizing the vesicles with 0.6% Na-Cholate before adding the protein at a 10:1 molar ratio (protein: liposome), giving a protein to lipid ratio of ca. 1:3500 in the outer monolayer. The detergent was then slowly removed by dialysis at 4 °C over night.

For generation of giant unilamellar vesicles (GUVs), a 1 mM stock solution of DOPC supplemented with 1% DPPE-biotinyl (2-dioleoyl-sn-glycero-3-phosphoethanolamine-N-biotinyl) (Avanti Polar Lipids) was used according to the procedure described in^42^. *c*NOR labelled with ATTO 594 (see below) was reconstituted into the GUVs using a mild detergent treatment with DDM; the protein solution containing 1 mM DDM was mixed with 20 μl GUV-solution to a final concentration of 0.05–0.25 μM protein and 0.05 mM DDM and incubated at room temperature for 30 min. The proteo-GUVs were then diluted 20 times in a 100 mM buffered glucose solution (10 mM phosphate buffer pH 7.4, 2 mM KCl) and transferred to a LabTek microscope chamber coated with streptavidin and further incubated at room temperature for 2 h. The dilution gave a final detergent concentration of 2.5 μM DDM in the sample.

### Steady-state activity measurements

The interaction between *c*NOR and *cd*_1_NiR was investigated by studying the multiple turnover activity of *c*NOR, either in detergent (0.05% DDM) or incorporated in vesicles, using a Clark-type electrode (World Precision Instruments, WPi) as in^18^. Briefly, the activity was measured in 50 mM HEPES at pH 7.0 with 50 mM KCl at room temperature. The buffer in the reaction chamber (total volume = 1 ml) was made anaerobic by adding the glucose (30 mM)/glucose oxidase (1 U/ml)/catalase (20 U/ml) system. Substrates were added with a syringe in the following order, horse heart (*hh*) cyt. *c* (varying concentrations), TMPD at varying concentrations, 5 equal additions of 10 µM NO (from NO-saturated water), and 3 mM sodium ascorbate. *c*NOR was added at various concentrations (20–80 nM) either prior to (*c*NOR in vesicles) or after all substrate additions (detergent solubilized). *cd*_1_NIR was added prior to the addition of NO, when specified. The data was recorded with the LabScribe2 software (WPi), and the maximum NO-reduction rate was calculated (at ~5 µM NO).

### Fluorescence labelling

*cd*_1_NiR and *c*NOR were fluorescently labelled using amino-reactive dyes. The protein concentration was set to 3 mg/ml and a 1/20 volume of NaHCO_3_ (pH 9.0) was added. *cd*_1_NiR was labelled with a 5-fold molar excess of Abberior STAR 635 (Abberior GmbH) and *c*NOR was labelled with a 3-fold molar excess of ATTO 594 (ATTO Tec GmbH) by incubating at room temperature while gently shaking for 1.5 h. Unbound dye was removed using a PD-10 column (GE Healthcare), equilibrated with a 10 mM phosphate buffer (pH 7.4) supplemented with 100 mM sucrose, 2 mM KCl and 1 mM (~0.05%) DDM.

### Fluorescence correlation spectroscopy (FCS) measurements and analyses

FCS measurements were performed on an instrument from Abberior Instruments (Göttingen, Germany), built on a stand from Olympus (IX83), and modified for two-color imaging (see^42^ for a detailed desciption of the experimental set up). Two fiber-coupled, pulsed (20 MHz) diode lasers emitting at 637 nm (PicoQuant AG, Berlin) and 594 nm (Abberior Instruments) were used for excitation, with the excitation pulses of the two lasers out of phase, to minimize cross-talk and enable fluorescence cross correlation of *cd*_1_NiR-STAR 635 on the membrane surface of the GUVs containing *c*NOR-ATTO 595. For details on the correlation and cross correlation analysis, see^42,43^.

The diffusion time of *cd*_1_NiR-STAR 635 (5 nM) was determined with FCS in the presence of increasing concentration of LUVs, with or without reconstituted *c*NOR. Normalized autocorrelation curves of the recorded fluorescence intensity fluctuations, G(τ), were calculated using a MatLab script, and the recorded *G*(*τ*) curves were then fitted using a model for 3D-diffusion, including two diffusional components and a population of a non-fluorescent triplet state (*T*) with a relaxation time *τ*_T_:

1 $$G(\tau )=\frac{1}{N(1-T)}\times a[{[1+\frac{\tau }{{\tau }_{D1}}]}^{-1}{[1+\frac{\tau }{{\beta }^{2}{\tau }_{D1}}]}^{-1/2}]+b[{[1+\frac{\tau }{{\tau }_{D2}}]}^{-1}{[1+\frac{\tau }{{\beta }^{2}{\tau }_{D2}}]}^{-1/2}]\times [1-T+T{e}^{-\frac{\tau }{{\tau }_{T}}}]$$

Here, *τ*_D1_ is the diffusion time of free *cd*_1_NiR-STAR 635 and *τ*_D2_ is the diffusion time of LUV-bound *cd*_1_NiR-STAR 635. β = ω_2_/ω_1,_ where ω_2_ and ω_1_ denote the 1/e^2^ extension of the FCS detection volume in along and perpendicular to the excitation beam direction, respectively. *N* is the average number of fluorescent molecules in the detection volume, and *a* and *b* the fractions of fluorescent molecules belonging to each of the two different diffusion components (with *a* + *b* = 1) The amplitudes of the component with diffusion time *τ*_D2_ were fitted with the binding model:

2 $${F}_{m}=\frac{{F}_{m}^{{\rm{\max }}}\times {c}_{{\rm{sol}}}}{{K}_{D}+{c}_{{\rm{sol}}}}$$

where *F*_m_ represents fraction of liposomes bound to *cd*_1_NiR-STAR 635, *c*_sol_ represents non-bound liposomes (plotted as number of free lipids), and *K*_D_ is the binding constant defined by the concentration at which half of the liposomes are bound (*F*_m_ = 0.5). The value of ${F}_{m}^{max}$ was set to unity.

## Supplementary information

Supplementary Information
