# Supplementary material for: Functional interactions between nitrite reductase and nitric oxide reductase from Paracoccus denitrificans
Source: Sci Rep. 2019 Nov 21;9:17234. doi: 10.1038/s41598-019-53553-z (PMC6872814; doi:10.1038/s41598-019-53553-z)
Supplement: Supplementary file 1 — Supplementary Information [file 41598_2019_53553_MOESM1_ESM.pdf]

Supplementary material for

Functional interactions between nitrite reductase and nitric oxide reductase from *Paracoccus denitrificans*

Ingrid Albertsson[1], Johannes Sjöholm[1,2], Josy ter Beek[1,3], Nicholas J. Watmough[4], Jerker Widengren[2], and Pia Ädelroth[1]*

[1]Department of Biochemistry and Biophysics, Stockholm University, Svante Arrhenius väg 16C, SE-106 91 Stockholm, Sweden

[2]Experimental Biomolecular Physics, Department of Applied Physics, Royal Institute of Technology (KTH), SE-106 91 Stockholm, Sweden.

[3]Current address: Department of Medical Biochemistry and Biophysics, Umeå University, Umeå SE-90187, Sweden.

[4]School of Biological Sciences, University of East Anglia, Norwich Research Park, Norwich NR4 7TJ UK.

*corresponding author: pia.adelroth@dbb.su.se

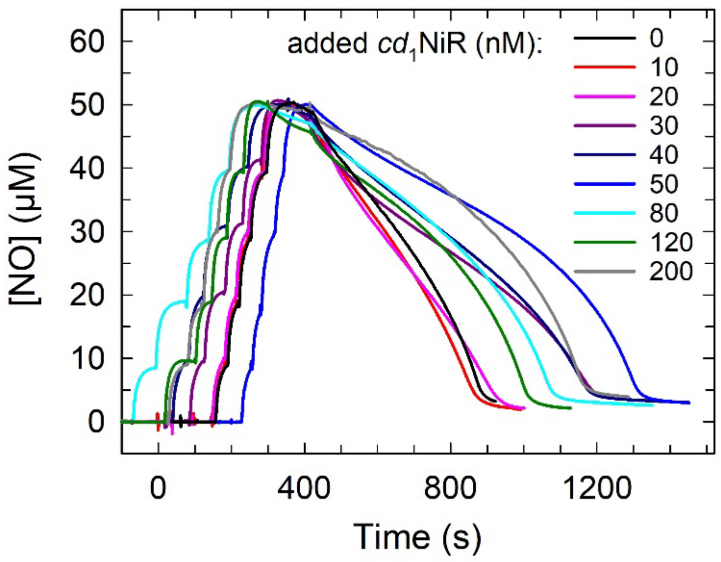

Supporting Figure 1.

Catalytic NO-reduction by *c*NOR with different amounts of *cd*1NiR (as indicated) added in the presence of the electron donors/mediators ascorbate, TMPD and cyt. *c*. After anaerobicity was reached in the chamber, the following additions were made (in order): cyt. *c*, TMPD (0.5 mM), *cd*1NiR, NO (5x10 µM), ascorbate (3 mM) and *c*NOR (40 nM). The traces are aligned to coincide at the point when *c*NOR was added for easier comparison. Experimental conditions: 50 mM HEPES, 50 mM KCl, 0.05 % DDM, pH 7.0, 30 mM glucose, 20 U/ml catalase, 1 U/ml glucose oxidase.

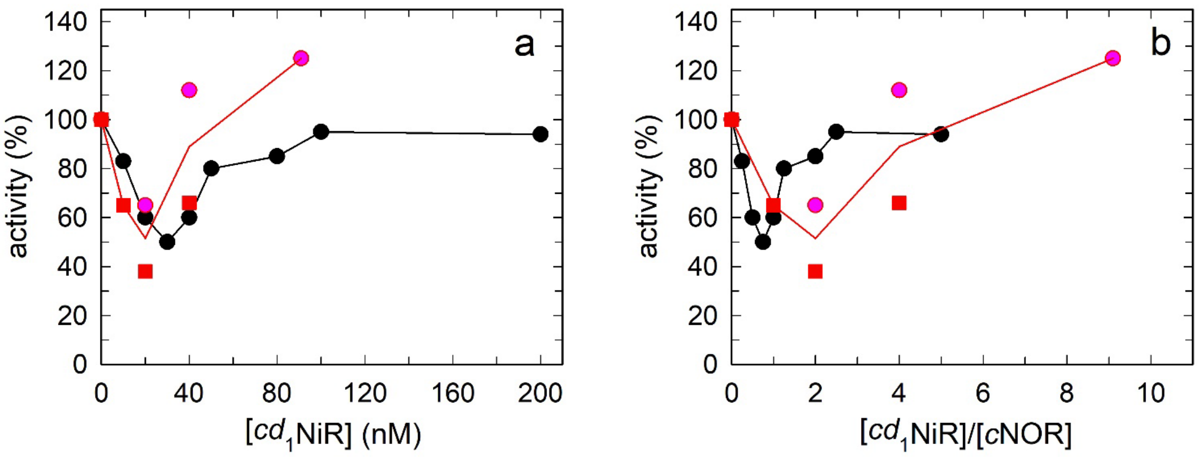

Supporting Figure 2.

A) Comparison between the effect of cd_1NiR on *c*NOR activity in detergent (black circles, same data as in **Fig. 2B**) and on liposome-reconstituted *c*NOR (at 10 nM, with cyt. *c* (pink circles), without cyt. *c* (red squares)). The red line is the average of the two data sets in liposomes (when they were done at the same [cd_1NiR]). The starting activity (with no added cd_1NiR) is set to 100%. **B)** The same plot as in **A)** but with the ratio cd_1NiR/*c*NOR rather than the cd_1NiR concentration as the x-axis. Note that this makes a substantial difference, possibly due to cd_1NiR interacting also with the liposomes. Experimental conditions as in Supporting Fig. 1, except that for the liposomes, no DDM was present and they were added before the addition of NO.

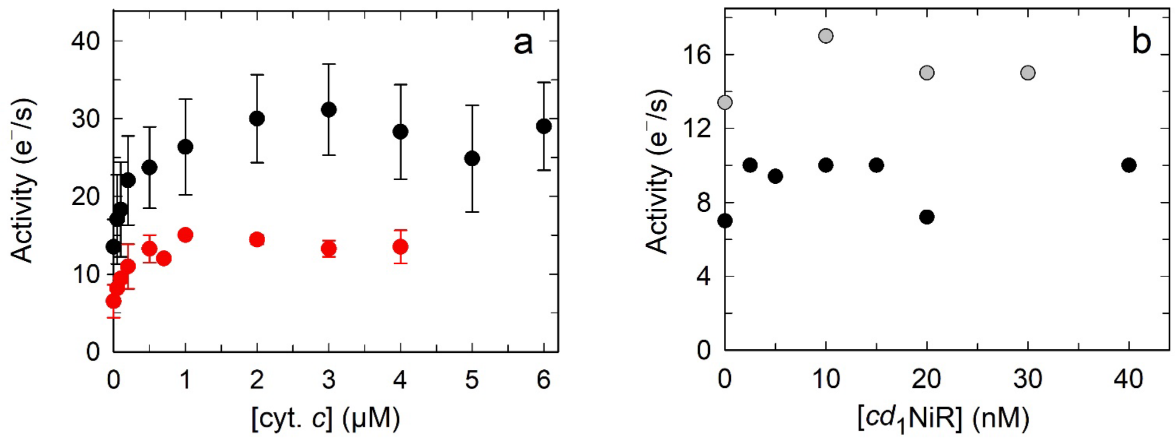

Supporting Figure 3.

A) Data from **Fig. 2B**, but plotted without subtracting the 'background' activity with no cyt. *c* added from each value. **B)** The (lack of) effect of cd_1NiR (at varied concentrations) on NO-consumption by *c*NOR in the absence of TPMD. Experimental conditions the same as in Supporting Fig. 1 except the omission of TMPD, and that either 5 µM (black circles), or 10 µM (grey circles) cyt. *c* was used.

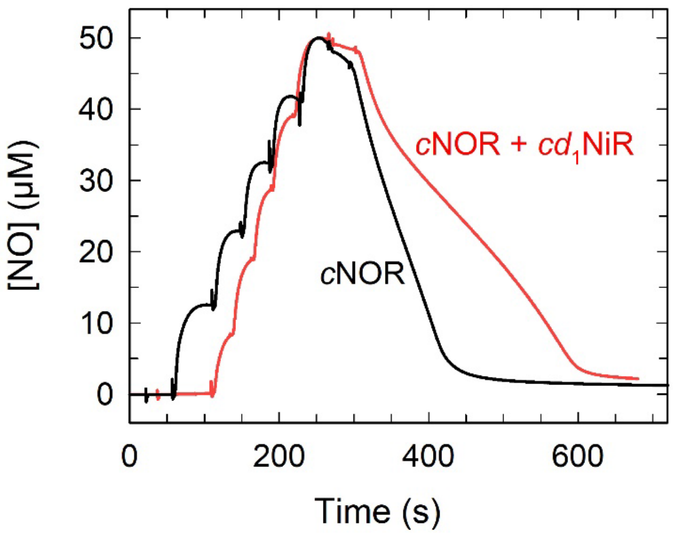

Supporting Figure 4.

Effect of *cd*$_1$NiR (red trace) on NO consumption catalyzed by *c*NOR (alone, black) using PMS as electron mediator instead of TMPD. The same two effects; lowering of maximum rate and changing the pattern of substrate inhibition as with TMPD are seen. Experimental conditions: Same buffer as in Supporting Fig. 1, 40 nM *c*NOR, 40 nM *cd*$_1$NiR, ascorbate (3 mM) and PMS (10 μM).

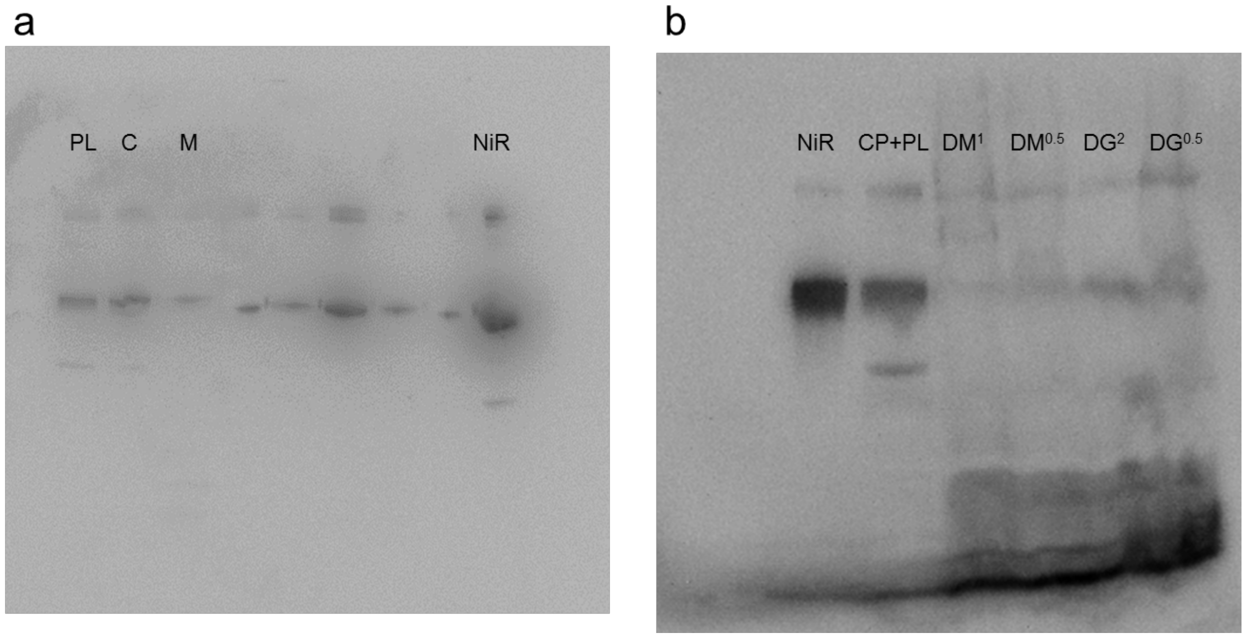

Supporting Figure 5:

Localization of cd_1NiR in the *P. denitrificans* cells using Western blot analysis. **A)** Fractions (20 µg) from anaerobically grown *P. denitrificans* cells were extracted, run on a 4-12 % precast gel, using MOPS (50 mM MOPS, 50 mM Tris Base, 0.1% SDS, 1 mM EDTA, pH 7.7) as running buffer. PL=periplasm, C=whole cells, M=membrane (in 0.5% DDM), NIR= purified cd_1NiR. Densitometry analysis indicated that compared to the periplasm (set to 100%), roughly 30 % cd_1NiR is associated with the membrane. **B)** Membrane fractions were solubilized in either digitonin (0.5-2 %) or DDM (0.5-1 %) with 50 mM KCl. NiR= purified cd_1NiR, CP+PL= cytoplasm plus periplasm, DM^1= membrane fraction solubilised in 1% DDM, $DM^{0.5}$= as DM^1 but in 0.5% DDM, DG^2= membrane fraction solubilised in 2% digitonin, $DG^{0.5}$= as DG^2 but in 0.5% digitonin. Note that comparisons should only be made between bands on each gel and not between the two gels.

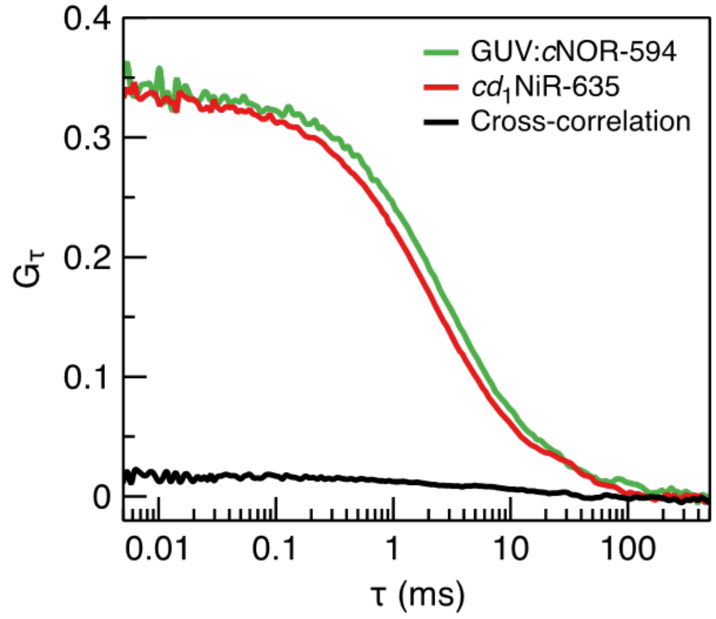

Supporting Figure 6.

Fluorescence cross correlation analysis of *c*NOR reconstituted in the GUV-membrane and *cd*$_1$NiR in solution. *c*NOR labelled with ATTO 594 was reconstituted in GUVs made of 99% DOPC and 1 % DPPE-biotin. A small cross correlation amplitude (< 7%) was obtained indicating no significant co-localization of *c*NOR and *cd*$_1$NiR in/at the membrane surface.

```
Paer |P24474|  78 VLRKGATGKPLTPDITQ-------------QRGQQYLEALITYGTPLGMPNWGSSGELS 124
Tthe |D5GU64|  47 VLRKGATGPALDPKKMA-------------ERGVEYLKAVIFGGLPGGMPDWGRQGILSE 93
Pstu |P24040|  53 VLRKGATGKNLEPHWSKTEADGKKTEGGTLNLGTKRLENIIAYGTEGGMVNYD--DILTK 110
Pstu2|F8H0E7| 107 VLRKGATGKPLTPDITQ-------------ERGQAYLEALITYGSPAGMPNWGTSNALTK 153
Pden |Q51700| 100 VLRKGATGKALTPDLTR-------------DLGFDYLQSFITYGSPAGMPNWGTSGELTA 146
Ppan |P72181| 100 VLRKGATGKALTPDLTR-------------DLGFDYLQSFITYGSPAGMPNWGTSGELSA 146
```

Supporting Figure 7.

Sequence alignment for *cd*$_1$NiRs around the area (78-124) that holds the Arg (R-96, marked in red) that is involved in complex formation with *c*NOR in the *Ps. aeruginosa* co-complex [1]. The different sequences are from *Ps. aeruginosa*, uniprot: P24474 (Paer), *Thermus thermophilus*, uniprot*:* D5GU64 (Tthe), *Pseudomonas stutzeri*, uniprot: P24040 (Pstu) and F8H0E7 (Pstu2), *P. denitrificans*, uniprot : Q51700 (Pden), *P. pantotrophus*, uniprot: P72181 (Ppan). Note that the corresponding residue in *P. denitrificans, P. pantotrophus* as well as one of the *P. stutzeri* sequences is a leucine. The alignment was made with Clustal Omega.

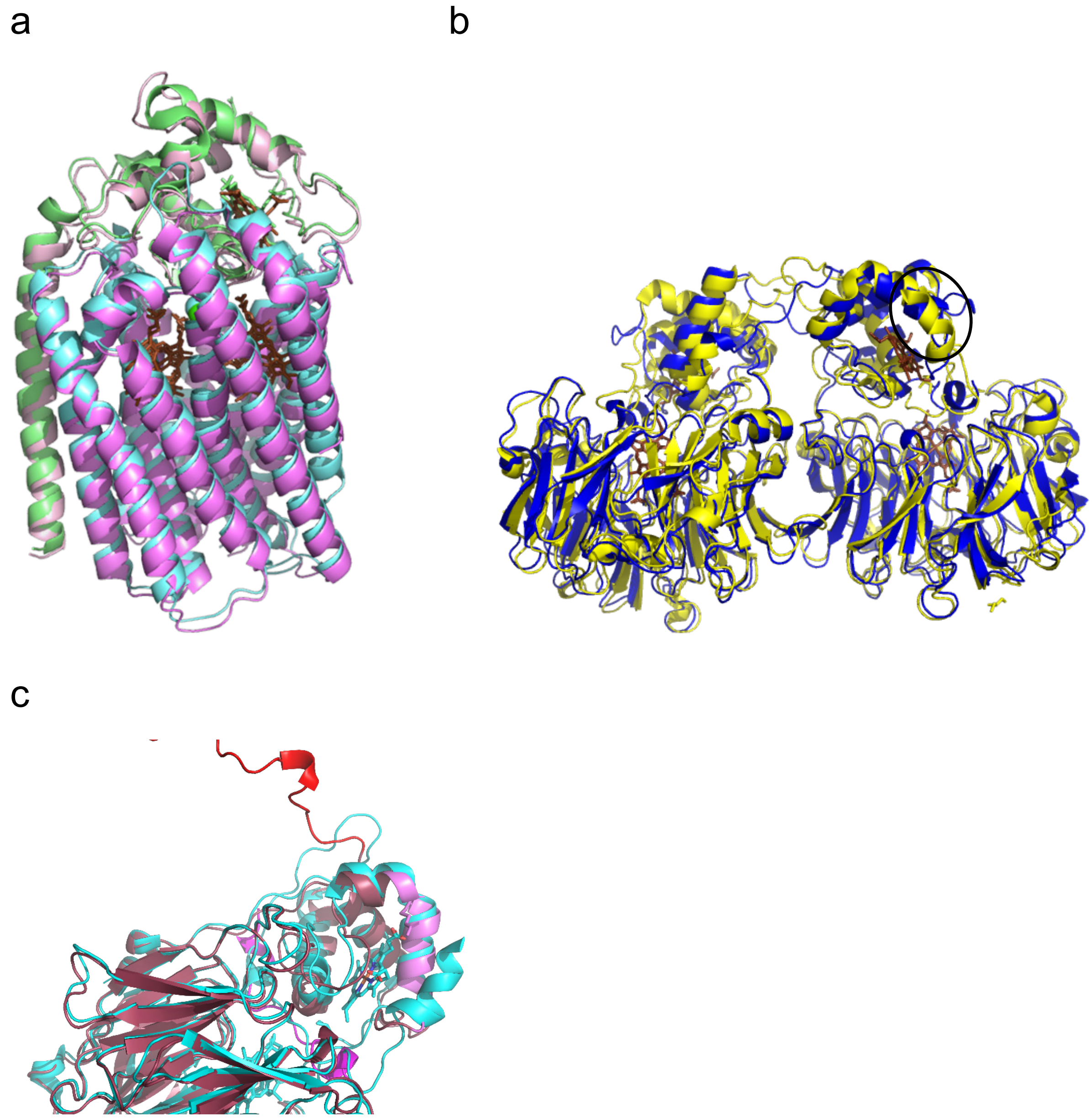

Supporting Figure 8.

A) Alignment of the *c*NOR structure from *P. aeruginosa* (PDB ID: 3O0R [2], NorC in green, and NorB in light blue) and the model of the *P. denitrificans* *c*NOR (NorC in pink, NorB in magenta). The overlap in the area that interacts with cd_1NiR is good. **B)** Alignment of the structures of cd_1NiR from *P. pantotrophus* (PDB ID: 1QKS [3], yellow) and from *Ps. aeruginosa* (PDB ID: 1NIR [4], blue). Note the 'extra' helix (circled) in the *c* domain in the *P. pantotrophus* structure. **C)** Zoom-in on the cd_1NiR overlay in the area interacting with *c*NOR. The figure shows the *P. pantotrophus* cd_1NiR (PDB ID:1QKS) in cyan, and the *P. aeruginosa* cd_1NiR (PDB ID: 1NIR) in dark pink, with the very end N-terminal (that is involved in domain swapping) in red/magenta for 1NIR. For the *P. pantotrophus* cd_1NiR it is the very N-terminal that forms the 'extra' helix. The helix that interacts with NorC in the co-complex plus the Arg-71 in *Ps. aeruginosa* cd_1NiR are highlighted in light pink.

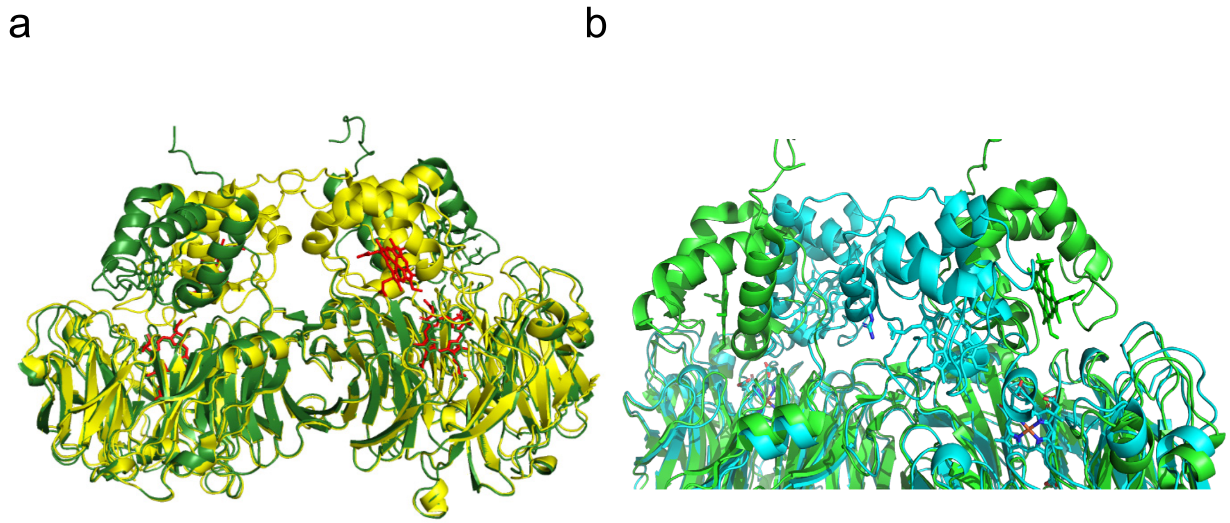

Supporting Figure 9.

A) The structure of *cd*$_1$NiR from *P. pantotrophus* (yellow) in oxidized form (PDB ID:1QKS, yellow) and reduced from (PDB ID: 1H9X [5], green). Note that there is very little overlap for the heme *c* domains. **B)** The *P. aeruginosa cd*$_1$NiR with R-71 highlighted in cyan, with the reduced *cd*$_1$NiR from *P. pantotrophus* in green. As is seen, there is no (or very little) overlap of the two *c* domains.

REFERENCES

1 Terasaka, E. *et al.* Dynamics of nitric oxide controlled by protein complex in bacterial system. *Proc. Natl. Acad. Sci. USA* **114**, 9888-9893, doi:10.1073/pnas.1621301114 (2017).

2 Hino, T. *et al.* Structural basis of biological N2O generation by bacterial nitric oxide reductase. *Science* **330**, 1666-1670 (2010).

3 Fulöp, V., Moir, J. W., Ferguson, S. J. & Hajdu, J. The anatomy of a bifunctional enzyme: structural basis for reduction of oxygen to water and synthesis of nitric oxide by cytochrome cd1. *Cell* **81**, 369-377 (1995).

4 Nurizzo, D. *et al.* N-terminal arm exchange is observed in the 2.15 A crystal structure of oxidized nitrite reductase from Pseudomonas aeruginosa. *Structure* **5**, 1157-1171 (1997).

5 Sjögren, T. & Hajdu, J. The Structure of an alternative form of Paracoccus pantotrophus cytochrome cd(1) nitrite reductase. *J. Biol. Chem.* **276**, 29450-29455, doi:10.1074/jbc.M103657200 (2001).
